# Temporal Changes in Tick‐Borne Pathogen Prevalence in Questing *Ixodes ricinus* Across Different Habitats in the North‐Eastern Italian Alps

**DOI:** 10.1002/mbo3.70010

**Published:** 2024-12-10

**Authors:** Fausta Rosso, Giulia Ferrari, Tobias Weil, Valentina Tagliapietra, Giovanni Marini, Francesca Dagostin, Daniele Arnoldi, Matteo Girardi, Annapaola Rizzoli

**Affiliations:** ^1^ Fondazione Edmund Mach, Research and Innovation Centre Trento Italy; ^2^ NBFC, National Biodiversity Future Center Palermo Italy

**Keywords:** habitat type, Italian Alps, *Ixodes ricinus*, spatio‐temporal scales, tick‐borne pathogens, zoonoses

## Abstract

Changes in land use, climate, and host community are leading to increased complexity in eco‐epidemiological relationships and the emergence of zoonoses. This study investigates the changes in the prevalence of several *Ixodes ricinus*‐transmitted pathogens in questing ticks over a 10‐year interval (2011–2013, 2020) in natural and agricultural habitats of the Autonomous Province of Trento (North‐eastern Alps), finding an average prevalence of infection of 27.1%. Analysis of 2652 ticks, investigating four infectious agents (*Borrelia burgdorferi* sensu lato, *Anaplasma* spp., *Rickettsia* spp., and *Babesia* spp.), revealed the circulation of 11 different zoonotic pathogens, with varying infection rates across different years and habitats. In 2020, we found a decrease in *Anaplasma phagocytophilum*, associated with agricultural habitats, and *Rickettsia* spp., found in all habitats. In the same year, *Babesia* spp. increased in both habitats, similar to *Borrelia burgdorferi* sensu stricto, which was related to natural habitats. Co‐infections were identified in 8% of positive‐tested ticks with different spatiotemporal associations, primarily in natural settings. Our results provide new evidence that the risk of infection with tick‐borne pathogens in the Alpine region varies over time and in different environments, broadening the current information on co‐infection rates and the circulation of zoonotic pathogens, previously not reported in this area.

## Graphical abstract

This study examines the prevalence of tick‐borne pathogens in Ixodes ricinus ticks in the north‐eastern Italian Alps. It found an average infection rate of 27.1%, with 11 zoonotic pathogens identified, showing varying infection rates across different years and habitats. Notably, co‐infections were present in 8% of positive ticks, highlighting the dynamic risk of tick‐borne diseases in this region.

## 1 Introduction

*Ixodid* ticks are obligate hematophagous ectoparasites whose life cycle consists of three stages (larva, nymph, and adult) that feed on a broad range of domestic and wild vertebrate hosts, including humans. Among the so‐called “hard ticks” (Acari: Ixodidae), the castor bean tick *Ixodes ricinus* (Linnaeus, 1758) is one of the most widespread species in the Western Palearctic, being reported in many European countries (Medlock et al. 2013; European Centre for Disease Prevention and Control 2023), including the Alpine region and Italy (Capelli et al. 2012; Lommano et al. 2012; Garcia‐Vozmediano et al. 2020).

Beyond their eco‐parasitological role, ticks are competent vectors for a large variety of microorganisms that can cause diseases of medical and veterinary importance at the global level (Heyman et al. 2010). Climate and land use change, human population growth, agricultural and wildlife management, movements of animals and people, and biodiversity loss are known as the main ecological drivers contributing to the emergence and spread of tick‐borne diseases (TBDs) (Rizzoli et al. 2019). Moreover, socioeconomic changes may alter spatio‐temporal encounters between hosts, vectors, and pathogens, as well as modulate their ecological niche, community structure, and abundance (Gottdenker et al. 2014; Guo, Bonebrake, and Gibson 2019). The composition of the vertebrate community, including the abundance of competent host species is likely pivotal in determining the acarological hazard (LoGiudice et al. 2008; Levi et al. 2016). In particular, the competence of vertebrate hosts can vary in the quality of blood meals they provide for ticks (i.e., the probability of survival and molting of feeding ticks), their attractiveness to vectors, and their ability to acquire or transmit infections (Ostfeld and Keesing 2012; Keesing and Ostfeld 2021).

Disease severity in hosts can also be driven by the coexistence of multiple pathogens, which are present within the same tick vector (co‐infection) (Cutler et al. 2021). Furthermore, the interaction among different pathogens can positively (facilitating/increasing) or negatively (competing/extinguishing) affect the emergence or successful coexistence of diseases within their ecological niche (Diuk‐Wasser, Vannier, and Krause 2016). Therefore, co‐infections of different microbes add another layer of complexity to the pathogen‐tick‐host relationship, providing relevant implications for public health authorities, especially in areas such as the Alps, where zoonotic spillover is very likely.

Our study site is located in the north‐eastern Italian Alps, in the Autonomous Province of Trento where warmer temperatures, increasing urbanization, high exploitation of touristic and recreational activities, and intensification of agriculture are enhancing the interactions among humans, wildlife, and vectors (Gössling 2002; Gobiet et al. 2014; Bebi et al. 2017). In this area, several studies have been conducted to unravel the ecological mechanisms driving the risk of emergence of tick‐borne zoonoses particularly those transmitted by *I. ricinus*. For instance, the incidence of tick‐borne encephalitis virus (TBEv), a flavivirus endemic in the area (Alfano et al. 2020), has increased as a consequence of a combination of climatic, environmental, and host‐related variables, which affects the number of co‐feeding ticks per host (Cagnacci et al. 2012; Collini et al. 2016; Rosà et al. 2019). The contribution of host assemblage in affecting tick infection rates has been confirmed by other studies on an obligate intracellular tick‐borne bacteria, *Anaplasma phagocytophilum*. In particular, both the bank vole (*Clethrionomys glareolus*) and the roe deer (*Capreolus capreolus*) emerged as competent reservoirs due to their potential capacity to infect *I. ricinus* larvae (Beninati et al. 2006; Carpi et al. 2009; Rosso et al. 2017; Baráková et al. 2018).

Since the Trentino‐Alto Adige region is considered a hotspot for tick‐borne disease circulation, the identification of the various pathogens carried by *Ixodes ricinus*, the assessment of the infection rate with each single pathogen including co‐infection, and the understanding of the factors affecting tick‐borne disease risk is essential for the implementation of public health intervention, including the most appropriate diagnostic protocols. This is particularly true for Alpine areas due to their popularity for outdoor activities (e.g., tourism, agricultural, silvicultural, and farming practices) and sensitivity to global changes. Indeed, both land use, wildlife management, and habitat fragmentation can alter disease risk and human exposure overtime (Millins et al. 2018; Dagostin et al. 2024) impacting host community composition and vector presence (Diuk‐Wasser, VanAcker, and Fernandez 2021; VanAcker et al. 2023). For instance, the prevalence of *Babesia* spp. and *Borrelia* (sin. *Borreliella*) *burgdorferi* sensu latu (s.l.) observed in questing *I. ricinus* ticks (Mantelli et al. 2006; Rosà et al. 2018) varied in relation to the habitat type.

In this study, we investigated the changes in prevalence and co‐occurrence of species belonging to *Anaplasma* spp., *B. burgdorferi* s.l., *Rickettsia* spp. and *Babesia* spp. in questing *I. ricinus* ticks over a 10‐year interval (2011–2013, 2020), in two different habitat types located in the north‐eastern Italian Alps.

## 2 Materials and Methods

### 2.1 Study Areas

**Figure 1:**
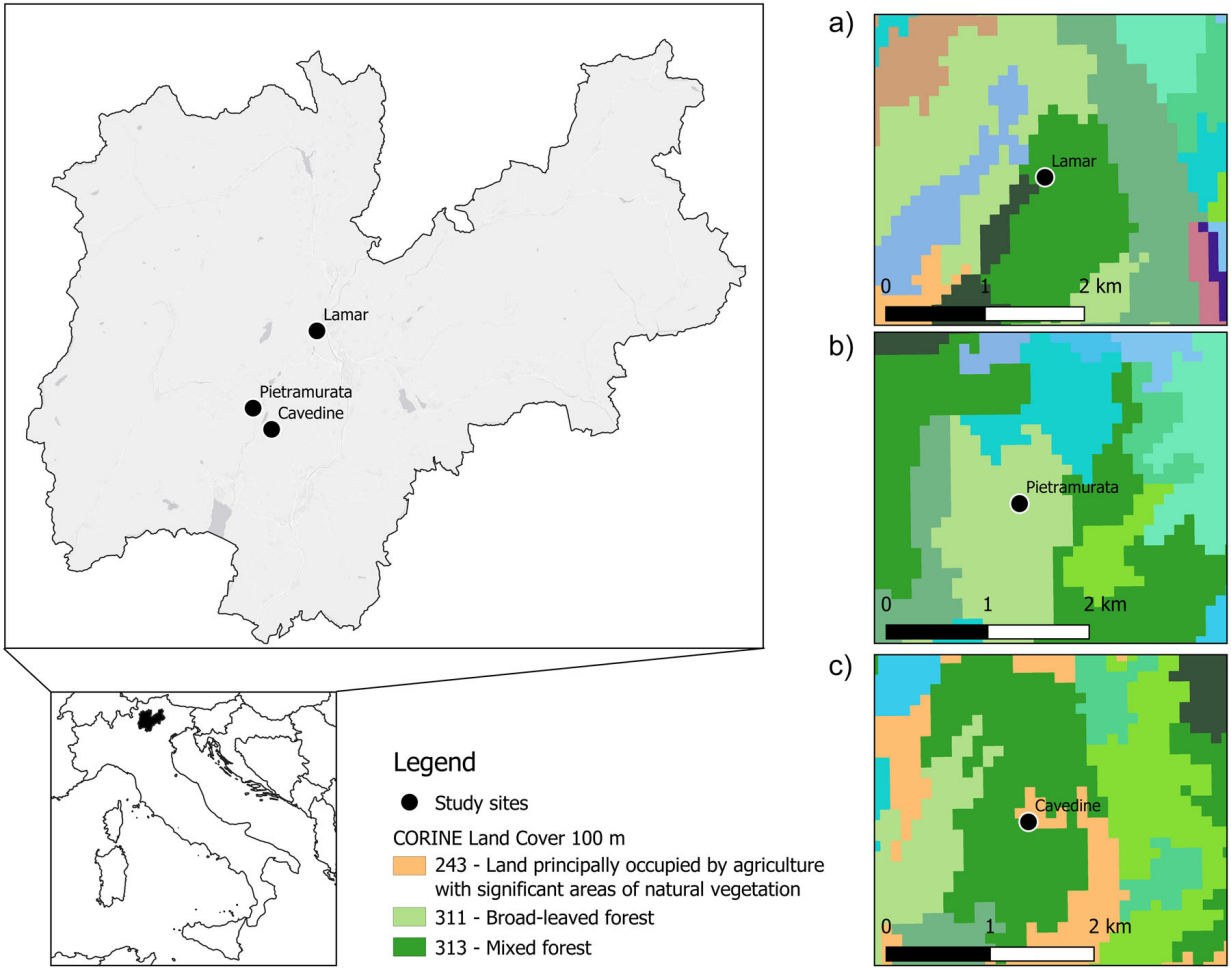
Map of the study area and locations of the sampling sites (black dots): (a) Lamar, (b) Pietramurata (monitored only in 2011), and (c) Cavedine. The right‐side panels show the different land covers of the three sites, according to the CORINE Land Cover classification (100‐m space resolution; European Environment Agency EEA 2018). https://land.copernicus.eu/pan-european/corine-land-cover/clc2018; accessed 7 September 2023).

The study sites are located in the Autonomous Province of Trento (north‐eastern Italian Alps) and are listed as Lamar (46.1249491–11.0630880, Vallelaghi municipality), Pietramurata (46.0150215–10.9257080, Dro municipality) and Cavedine (11.1755773–46.1259899, Cavedine municipality) (Figure 1). The altitude of the three sites spans from 600 to 800 m a.s.l. and they fall into the warm‐temperate alpine climate zone (sensu Köppen‐Geiger classification, Rubel et al. 2017), characterized by moderately cold winters and hot/warm summers (Meteotrentino, https://www.meteotrentino.it). Each study site is classified either as “natural“ or “agricultural“ according to CORINE Land Cover classification layers with a space resolution of 100 m (European Environment Agency EEA 2018). Specifically, Lamar and Pietramurata belong to categories 25 and 23, that is, mixed (broad‐leaved and coniferous) and broad‐leaved forest, respectively. Cavedine falls into category 21, that is, land principally occupied by agriculture with significant areas of natural vegetation. Following this classification, we defined Lamar and Pietramurata as natural habitats, while Cavedine as an agricultural one.

### 2.2 Questing Ticks Sampling

Questing ticks were collected in Cavedine and Lamar sites three times per year (April, May, June) in 2011, 2012, and 2013, while in 2020 sampling was performed only in June. The Pietramurata site was investigated only in 2011 (April, May, and June). For all years of sampling (2011, 2012, 2013, and 2020) standard dragging with 1‐square‐meter white flannel cloth was carried out through vegetation and leaf litter along transects of 100 m length. Tick nymphs and adults attached to the cloth were counted and collected in tubes, while larvae were discarded.

Each tick was then washed in ethanol 70%, rinsed with water for molecular biology, and dried with paper before the morphological identification at the species level using a stereomicroscope. All samples were stored at −80°C until DNA extraction.

### 2.3 Laboratory Analyses

Ticks were disrupted with TissueLyser II (Qiagen‐GmbH‐Hilden, Germany) at 30‐Hz frequency (corresponding to 1800 oscillations/minute) for 3.30 min, in single tubes using 100 µL of PBS (Phosphate Buffered Saline) and previously sterilized stainless‐steel beads (5 mm diameter). DNA was extracted from individual nymphs and adults using the Qiamp DNA Investigator kit (Qiagen‐GmbH‐Hilden, Germany) protocol “tissues” optimized for the Qiacube extractor. For samples collected in 2020, the same DNA extraction kit and protocol were used, but instead of using the automated protocol (Qiacube extractor), DNA was extracted manually following the manufacturer's instructions. The final elution volume was 60 µL for both nymphs and adults. DNA extracted from ticks was used for the detection of the following pathogen genera*: Anaplasma*, *Borrelia, Rickettsia*, and *Babesia*. For *Babesia* spp., a single‐step conventional PCR using genus‐specific primers targeting the 18S rRNA gene (Casati et al. 2006) was used, while for *Rickettsia* spp. the target was the 17‐ kDa surface antigen and a semi‐nested PCR was used (Reye et al. 2010). The amplification of the latter gene was not sufficient for distinguishing *Rickettsia* species in all samples, thus, we used a more variable gene coding for the outer membrane protein A (ompA). The analysis for *Borrelia* species was carried out by applying a nested PCR targeting the intergenic spacer codifying for ribosomal RNA 5S rRNA and 23S rRNA (modified from Rijpkema et al. 1995). Finally, for *Anaplasma* spp., a nested PCR amplification of the partial 16 s rRNA gene was applied (Massung et al. 1998). Temperature cycling profile and specific primers were described in Appendix A (see Tables A1, A2, A3).

Visualization of the success of amplification was confirmed using the Qiagen QIAxcel capillary electrophoresis system. Positive PCR products were purified enzymatically using ExoSAP‐IT (USB, Cleveland, OH, USA) according to the manufacturer's instructions and then sequenced using Sanger Sequencing (on an ABI 3730xl Genetic Analyzer, Sequencing Platform Fondazione Edmund Mach). The DNA sequences were compared with the data stored in the GenBank database with the Basic Local Alignment Search Tool (Blast; online version). Species‐specific alignments were built using the MUSCLE algorithm implemented in MEGA‐X (Kumar et al. 2018), manually curated, and deposited into the NCBI GenBank database.

In samples collected in 2020, we noticed some double peaks or dubious bases in electropherograms of sequences belonging to *B. burgdorferi* s.l. complex, which suggested a coinfection with different genotypes of this pathogen. For this reason, we applied a cloning procedure on seven samples using the TOPO XL PCR Cloning kit (Invitrogen, Life Technologies), and the extracted plasmid DNA was used for sequencing. The procedure was successful only in one sample in which we identified a coinfection (sample name: male tick from Lamar study site, M12).

### 2.4 Statistical Analyses

All statistical analyses were carried out using R V4.2.2 (R Core Team 2023), and package *tidyverse* (Wickham 2017) and *ggplot2* (Wickham 2016) were used for data management and graphics.

#### 2.4.1 Pathogen Community

With the term “pathogen community” we refer to the group of TBPs (tick‐borne pathogens, TBPs) (bacteria and protozoa) identified in the collected ticks. Species richness using the Shannon diversity Index (alpha diversity) was estimated for bacteria and protozoa found in different habitat types and across sampling years fitting the function *diversity* in the *vegan* package (Oksanen et al. 2016). Pathogen community composition was explored through an alluvial diagram using the *ggalluvial* package (Brunson and Read 2023), that expresses the descriptive associations among categorical variables. In our case, the alluvial diagram described the changes and trends of pathogens' infections across years and in different habitat types.

#### 2.4.2 Tick Infection Probability

**Figure B1:**
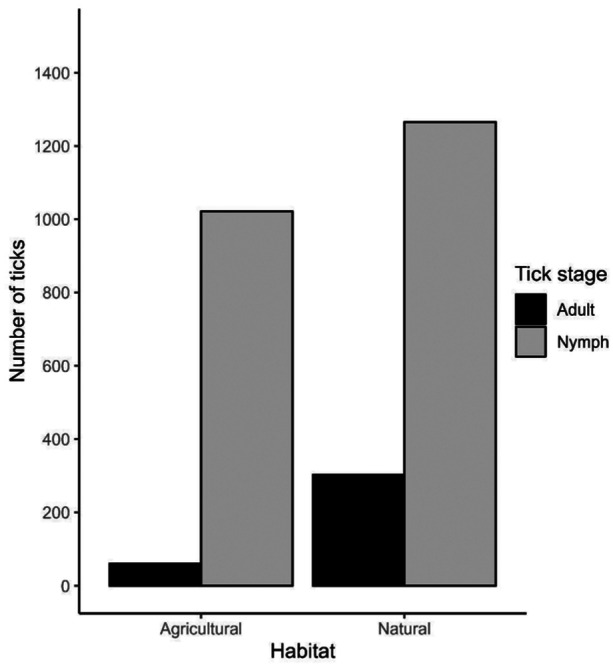
Total number of nymph and adult *Ixodes ricinus* ticks collected in the two habitat types (agricultural and natural; Province of Trento, Italy). GLMs with Poisson distribution; nymph: *β* = 0.21 ± 0.04; *p*‐value < 0.001; adult: *β* = 1.60 ± 0.14; *p*‐value < 0.001; reference category: agricultural habitat.

Prevalence of each TBP across years, habitat type, and tick stage was calculated in each year, habitat type and tick stage with a 95% confidence interval (CI), using the *EpiR* package (Carstensen et al. 2021). Two Proportion *Z*‐test was applied to analyze the differences in the prevalence rates of TBPs among habitat types and years. After testing the collinearity between habitat type and tick stage (Appendix B, Figure B1), univariate Generalized Linear Models (GLMs) with binomial error distribution were fitted to investigate how the proportion of ticks that were infected with each detected TBP (i.e., prevalence) varies depending on habitat type and tick stage (explanatory variables). The differences were considered statistically significant if the *p*‐values were < 0.05.

*Ggeffects* package (Lüdecke 2023) was used to retain model predictions and *sjPlot* (Lüdecke 2020) to create HTML tables from regression models.

## 3 Results

### 3.1 Ticks Screening

**Figure C1:**
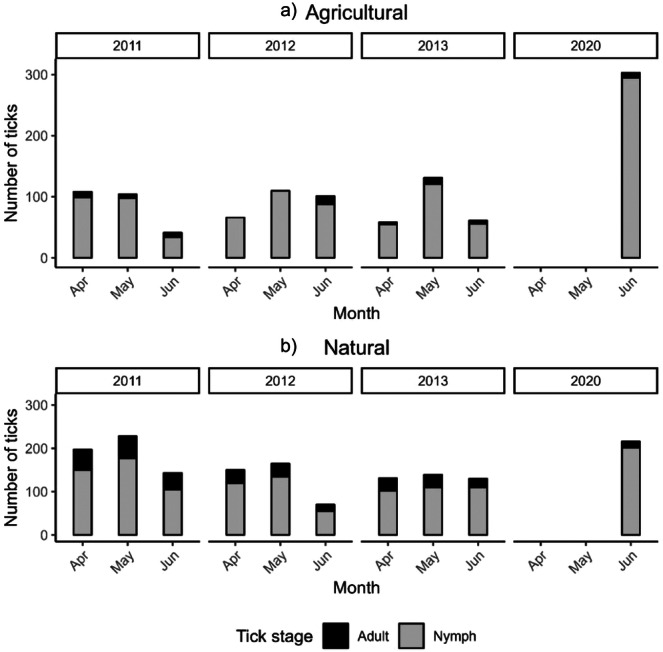
Total number of questing *Ixodes ricinus* ticks per life stage, sampling month and year, in two different habitats: Panel a: agricultural habitat and Panel b: natural habitat; (Province of Trento, Italy, 2011–2013; 2020).

A total of 2652 ticks (2288 nymphs and 364 adults) were collected in the study sites from 2011 to 2020 (821 in 2011, 662 in 2012, 650 in 2013, and 519 in 2020; Table 1). Given the recent identification of *I. inopinatus* in the Italian peninsula (Daněk et al. 2024), we did not exclude the presence of the species or its hybrids in the collected samples. In this sense, all the identified ticks are considered to belong to the *I. ricinus* complex that includes also *I. inopinatus*. When discriminating between habitat types, 1083 ticks were collected in the agricultural site (Cavedine), while 1569 in natural ones (Pietramurata and Lamar). Across all years, nymphs were the most abundant life stage (86.3%), while adults were in percentage more abundant in natural habitats than in agricultural ones (19.3% and 5.6%, respectively) (see also Figure C1b). Ticks collected in consecutive months (April, May, June) showed a peak in May both in natural and agricultural habitats.

**Table 1:** Tick‐borne pathogens prevalence across sampling years (2011, 2012, 2013, 2020), habitat type (agricultural and natural), and tick stage (adult, nymph) in the Province of Trento, Italy.

	2011		2012		2013		2020		
	Nymphs	Adults	Nymphs	Adults	Nymphs	Adults	Nymphs	Adults	Total
Habitat type Agricultural	*n* = 231	*n* = 22	*n* = 264	*n* = 13	*n* = 232	*n* = 18	*n* = 295	*n = 8*	1083
Anaplasma phagocytophilum	4 1.73% (0.47–4.37)	1 4.55% (0.12–22‐84)	5 1.89% (0.62–4.36)	1 7.69% (0.19–36.03)	8 3.45% (1.50–6.68)	2 11.11% (1.38–34.71)	3 1.02% (0.21–2.94)	0	**24**
Babesia divergens	0	0	—	—	—	—	1 0.34% (0.01–1.87)	0	**1**
**Babesia venatorum**	**3** **1.30% (0.27**–**3.75)**	**0**	**—**	**—**	**—**	**—**	**3** **1.02% (0.21–2.94)**	**0**	**6**
Borrelia afzelii	9 3.90% (1.80–7.27)	0	11 4.17% (2.10–7.33)	0	14 6.03% (3.34–9.92)	1 5.56% (0.14–27.29)	12 4.07% (2.12–7.00)	0	**47**
**Borrelia burgdorferi s.s.**	**6** **2.60% (0.96–5.57)**	**1** **4.55% (0.12–22.84)**	**2** **0.76% (0.09–2.71)**	**0**	**2** **0.86% (0.10**–**3.08)**	**0**	**11** **3.73% (1.88–6.57)**	**1** **12.50% (0.32–52.65)**	**23**
Borrelia garinii	11 4.76% (2.40–8.36)	0	24 9.09% (5.91–13.22)	1 7.69% (0.19–36.03)	11 4.74% (2.39–8.33)	1 5.56% (0.14–27.29)	12 4.07% (2.12–7.00)	1 12.50% (0.32–52.65)	**61**
**Borrelia lusitaniae**	**0**	**0**	**0**	**0**	**0**	**0**	**2** **0.68% (0.08–2.43)**	**0**	**2**
Borrelia valaisiana	8 3.46% (1.51–6.71)	0	7 2.65% (1.07–5.39)	1 7.69% (0.19–36.03)	13 5.60% (3.02–9.39)	1 5.56% (0.14–27.29)	10 3.39% (1.64–6.15)	1 12.50% (0.32–52.65)	**41**
Rickettsia helvetica	8 3.46% (1.51–6.71)	0	17 6.44% (3.80–10.11)	3 23.08% (5.04–53.81)	16 6.90% (3.99–10.96)	1 5.56% (0.14–27.29)	13 4.41% (2.37–7.42)	0	**58**
Rickettsia monacensis	9 3.90% (1.80–7.27)	0	5 1.89% (0.62–4.36)	0	11 4.74% (2.39–8.33)	0	6 2.03% (0.75–4.37)	0	**31**
Rickettsia slovaca	1 0.43% (0.01–2.39)	0	0	0	0	0	0	0	**1**
**Total positive ticks in agricultural habitat**	**59**	**2**	**71**	**6**	**75**	**6**	**73**	**3**	**295**

	2011		2012		2013		2020		
Habitat type	Nymphs	Adults	Nymphs	Adults	Nymphs	Adults	Nymphs	Adults	Total
Natural	*n* = 432	*n* = 136	*n* = 310	*n* = 75	*n* = 322	*n* = 78	*n* = 202	*n* = 14	1569
Anaplasma phagocytophilum	5 1.16% (0.38–2.68)	5 3.68% (1.20–8.37)	2 0.65% (0.08–2.31)	0	3 0.93% (0.19–2.70)	4 5.13% (1.41–12.61)	0	0	**19**
Babesia divergens	1 0.23% (0.01–1.28)	0	—	—	—	—	3 1.49% (0.31–4.28)	0	**4**
**Babesia venatorum**	**0**	**0**	**—**	**—**	**—**	**—**	**0**	**1** **7.14% (0.18–33.87)**	**1**
Borrelia afzelii	18 4.17% (2.49–6.51)	6 4.41% (1.64–9.36)	12 3.87% (2.02–6.66)	3 4.00% (0.83–11.25)	20 6.21% (3.83–9.43)	3 3.85% (0.80–10.83)	19 9.41% (5.76–14.30)	1* 7.14% (0.18–33.87)	**82**
**Borrelia burgdorferi s.s.**	**28** **6.48% (4.35–9.23)**	**6** **4.41% (1.64–9.36)**	**9** **2.90% (1.34–5.44)**	**5** **6.67% (2.20–14.88)**	**24** **7.45% (4.83–10.89)**	**3** **3.85% (0.80–10.83)**	**12** **5.94% (3.11–10.15)**	2* **14.29% (1.78–42.81)**	**89**
Borrelia garinii	31 7.18% (4.93–10.03	9 6.62% (3.07–12.19)	23 7.42% (4.76–10.92)	4 5.33% (1.47–13.10)	27 8.39% (5.60–11.97)	7 8.97% (3.68–17.62)	8 3.96% (1.73–7.65)	1 7.14% (0.18–33.87	**110**
**Borrelia lusitaniae**	**10** **2.31% (1.12–4.22)**	**5** **3.68% (1.20–8.37)**	**1** **0.32% (0.01–1.78)**	**0**	**1** **0.31% (0.01–1.72)**	**1** **1.28% (0.03–6.94)**	**1** **0.50% (0.01–2.73)**	**2** **14.29% (1.78–42.81)**	**21**
Borrelia valaisiana	15 3.47% (1.96–5.66)	5 3.68% (1.20–8.37)	23 7.42% (4.76–10.92)	3 4.00% (0.83–11.25)	19 5.90% (3.59–9.06)	6 7.69% (2.88–15.99)	12 5.94% (3.11–10.15)	0	**83**
Rickettsia helvetica	21 4.86% (3.03–7.33)	8 5.88% (2.57–11.26)	17 5.48% (3.23–8.64)	4 5.33% (1.47–13.10)	14 4.35% (2.40–7.19)	6 7.69% (2.88–15.99)	9 4.46% (2.06–8.29)	0	**79**
Rickettsia monacensis	7 1.62% (0.65–3.31)	6 4.41% (1.64–9.36)	12 3.87% (2.02–6.66)	6 8.00% (2.99–16.60)	16 4.97% (2.87–7.94)	2 2.56% (0.31–8.96)	3 1.49% (0.31–4.28)	1 7.14% (0.18–33.87)	**52**
Rickettsia slovaca	0	0	0	0	0	0	0	0	**0**
**Total positive ticks in natural habitat**	**136**	**50**	**99**	**25**	**124**	**32**	**67**	**8**	**541**
**Total positive ticks**	**195**	**52**	**170**	**31**	**199**	**38**	**140**	**11**	**836**

*Note:* Each cell includes: *n*. of positive samples, prevalence (%), and within brackets 95% confidence intervals. Legend: “–”: no screening performed; *: cloning sample, co‐infection of *Borrelia afzelii* and *B. burgdorferi* in the same tick sample. In bold are pathogens with significant statistical difference among habitat types (see Appendix E): *Babesia venatorum* (*Z*‐test, *p*‐value = 0.04), *B. burgdorferi* s.s. (*Z*‐test, *p*‐value = 1.253e‐05) and *Borrelia lusitaniae* (*Z*‐test, *p*‐value = 0.003).

### 3.2 Tick‐Borne Pathogens Community Composition

A total of 11 species belonging to four genera of microorganisms were found in the collected *I. ricinus* ticks (Table 1). In particular: *A. phagocytophilum*, *R. helvetica*, *R. monacensis, R. slovaca*, *B. burgdorferi* sensu strictu (s.s.), *B. afzelii*, *B. garinii*, *B. lusitaniae, B. valaisiana, Ba. divergens* and *Ba. venatorum* (formerly *Babesia* spp. *EU1*), all characterized by zoonotic relevance. They were recorded in both habitat types except for *R. slovaca* that was found only in the agricultural areas.

**Figure 2:**
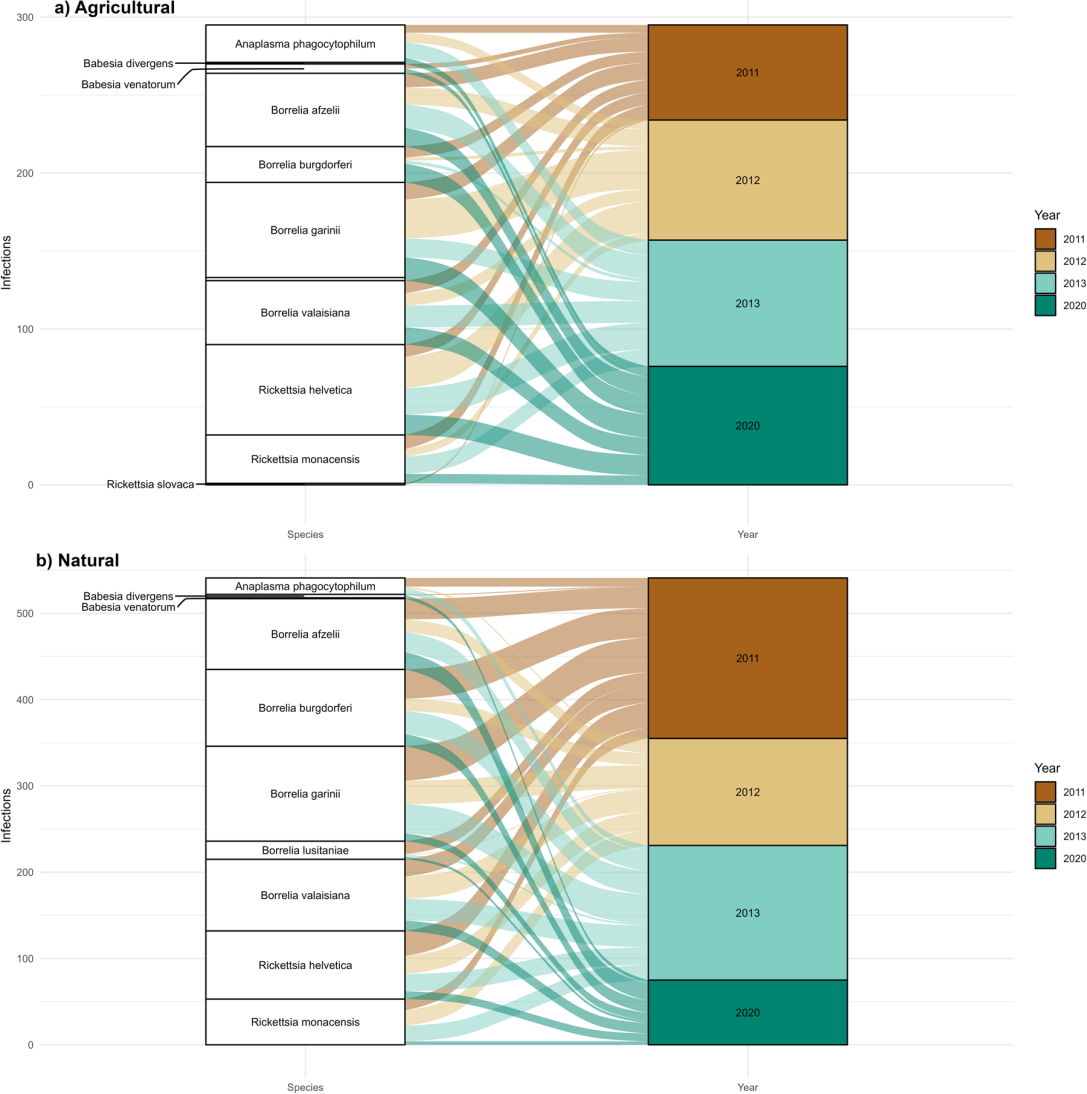
Alluvial diagram visualizing the changes in tick‐borne pathogens' infections across sampling years (2011, 2012, 2013, 2020; color gradient) and in different habitat types (Panel a: agricultural habitat; Panel b: natural habitat).

No differences in relative abundances of TBPs (Shannon Index) were observed between the agricultural and natural habitat types and across the sampling years (Table D1), although when these pathogens were represented *ensemble*, variations across years were evident in the tick‐borne pathogen community. Notably, qualitatively in the period 2011–2013 we observed an overall higher infections of TBPs in natural habitats, with respect to 2020 (Figure 2).

### 3.3 Tick Infection Probability

The overall number of infected ticks was 720, with 836 infections in total, and a total prevalence of 27.1% (720/2652). In particular, *B. burgdorferi* s.l., *Rickettsia* spp., *A. phagocytophilum*, and *Babesia* spp. were 21.1% (559/2652), 8.4% (222/2652), 1.6% (43/2652), and 0.4% (12/2652), respectively (for more details see Table 1).

**Figure 3:**
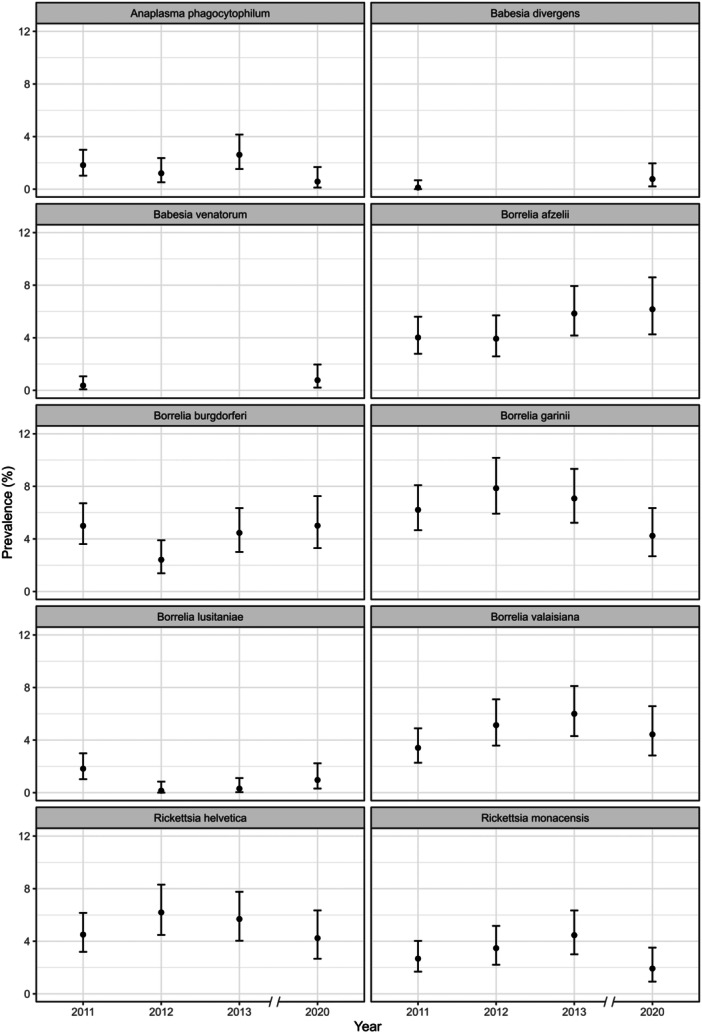
Prevalence rates for the tick‐borne pathogens investigated in *I. ricinus* ticks collected in the Province of Trento, Italy, across sampling years (2011–2013, 2020). Every facet shows one single pathogen species (*R. slovaca* is not shown in the figure since it was detected only in 2011). Vertical bars represent the 95% confidence interval.

**Figure E1:**
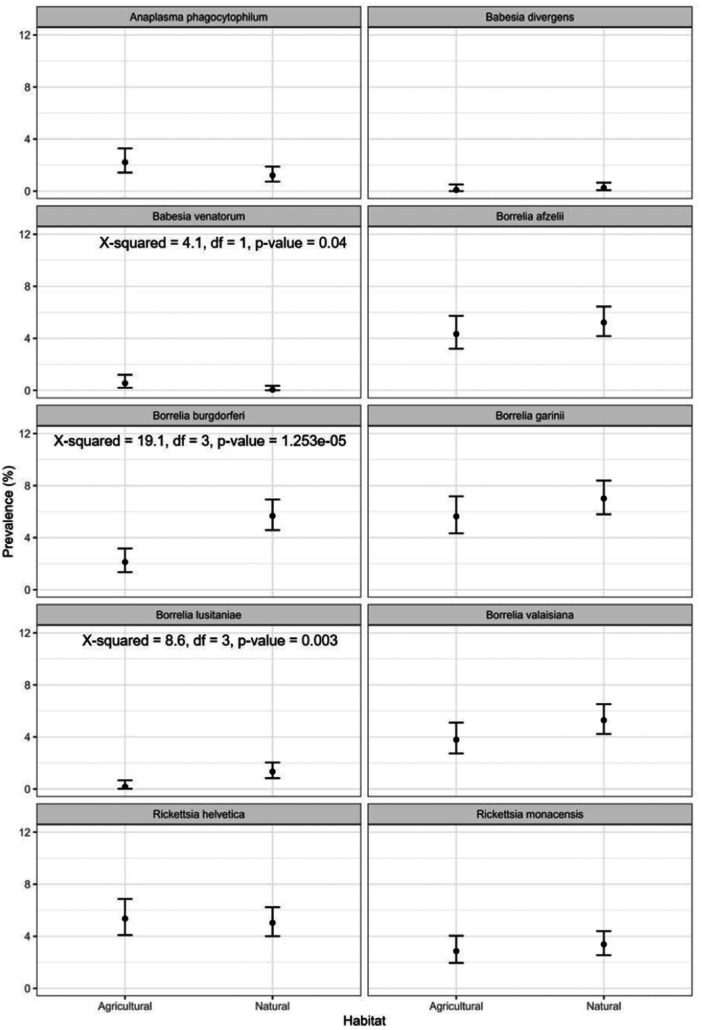
Prevalence rates for each of the tick‐borne pathogen investigated in *Ixodes ricinus* ticks collected across habitat types (agricultural and natural) (Province of Trento, Italy, 2011–2013, 2020). Vertical bars represent the 95% confidence interval: *Babesia venatorum* (*Z*‐test, *p*‐value = 0.04), *Borrelia burgdorferi s.s*. (*Z*‐test, *p*‐value = 1.253e^−05^) and *Borrelia lusitaniae* (*Z*‐test, *p*‐value = 0.003).

The prevalence between years varied depending on the specific TBP considered (see Figure E1, Appendix E). *A. phagocytophilum* showed a slight increase in 2013 and a statistically significantly lower prevalence in 2020 compared to previous years (*Z*‐test, *p‐*value = 0.03) (Figure 3). In contrast, *Ba. venatorum* and *Ba. divergens* had low prevalence in 2011, were not evaluated in 2012 and 2013, and increased significantly in 2020 (*Z*‐test, *p‐*value = 0.07 for *Ba. divergens* and *Z*‐test, *p‐*value = 0.03 for *Ba. venatorum*) (Figure 3). Among the five species of the *B. burgdorferi* s.l. complex, *B. burgdorferi* s.s. and *B. lusitaniae* showed a significant decrease in prevalence in 2012, and an increase in 2013 and 2020 (*Z*‐test, *p‐*value = 0.05 and *p‐*value = 0.001, respectively), while *B. afzelii*, *B. garinii*, and *B. valaisiana* did not show any significant variation (see Figure 3). Regarding *Rickettsia* spp., there were no significant variations in prevalence across the years, while *R. slovaca* was found only in 2011.

**Figure 4:**
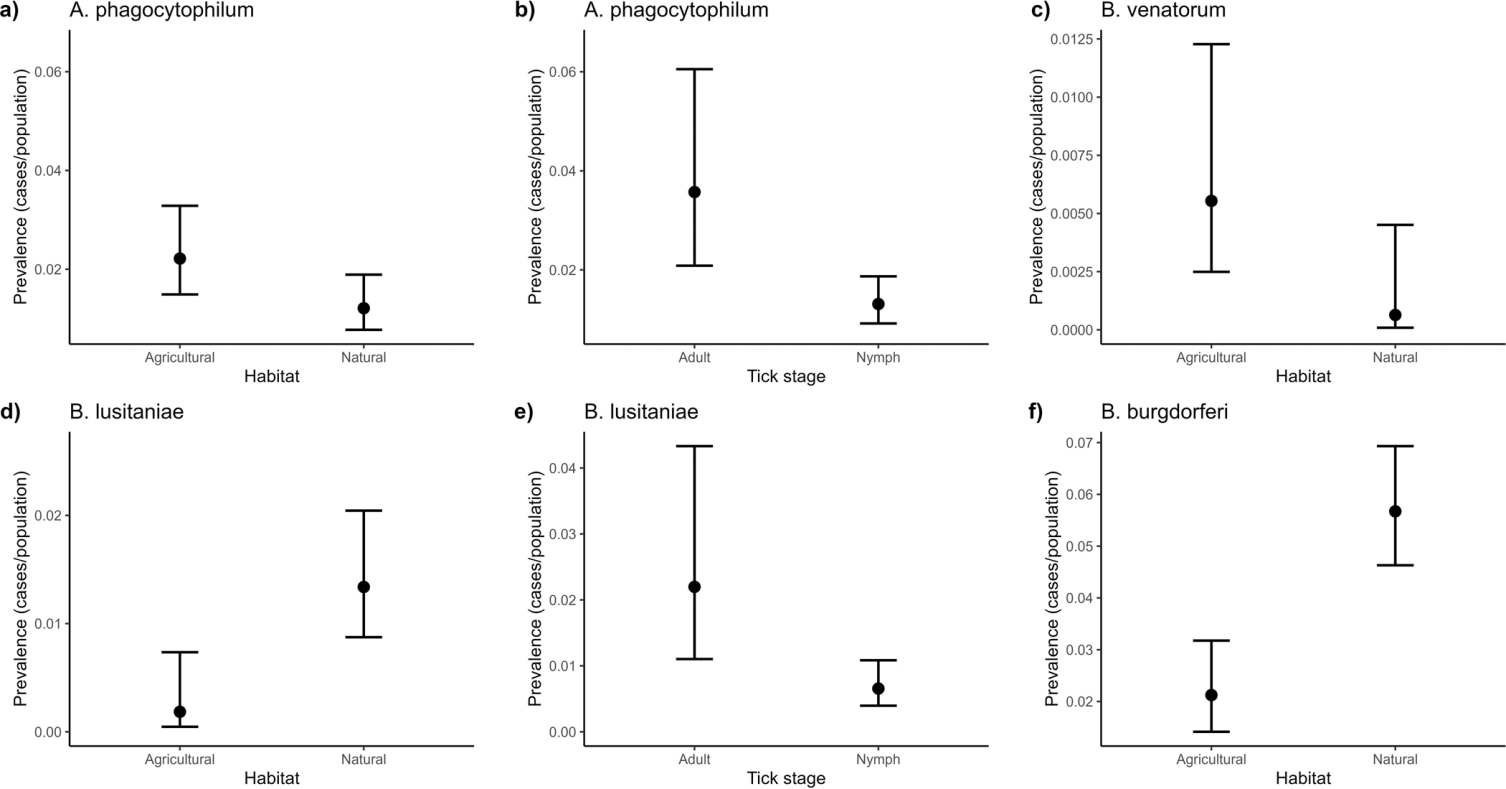
Relationships between the prevalence of tick‐borne pathogens, habitat types, and tick stages. Only statistically significant relationships are shown. Black dots are the average values of prevalence for each pathogen, while bars represent the 95% confidence intervals. Panels have different *Y*‐axis ranges to assist visualization. Panel a, b: A. phagocytophilum; Panel c: B. venatorum; Panel d, e: B. lusitaniae; Panel f: B. burgdorferi

Following the collinearity test, tick stage and habitat type were retained and modelled in the univariate Binomial GLMs (see Tables F1 and F2). In particular, the prevalence of *A. phagocytophilum* was lower in natural habitats (Figure 4a, see Table F1) and in nymphs (Figure 4b, see Table F2). *Ba. venatorum* decreased prevalence in natural habitats (Figure 4c, see Table F1), while the tick stage was not statistically significantly different. Among *B. burgdorferi* s.l. complex, only *B. lusitaniae* and *B. burgdorferi s.s*. showed statistically significant relationships: both species were more prevalent in natural habitats (Figure 4d,f, Table F1), while tick stage was relevant only for *B. lusitaniae* with a lower prevalence in nymphs (Figure 4e, see Table F2). Finally, the prevalence of *Rickettsia* spp. did not retain any significant association with tick stage or habitat type (Tables F1, F2 and F2).

### 3.4 Co‐Infections in Ticks

**Figure 5:**
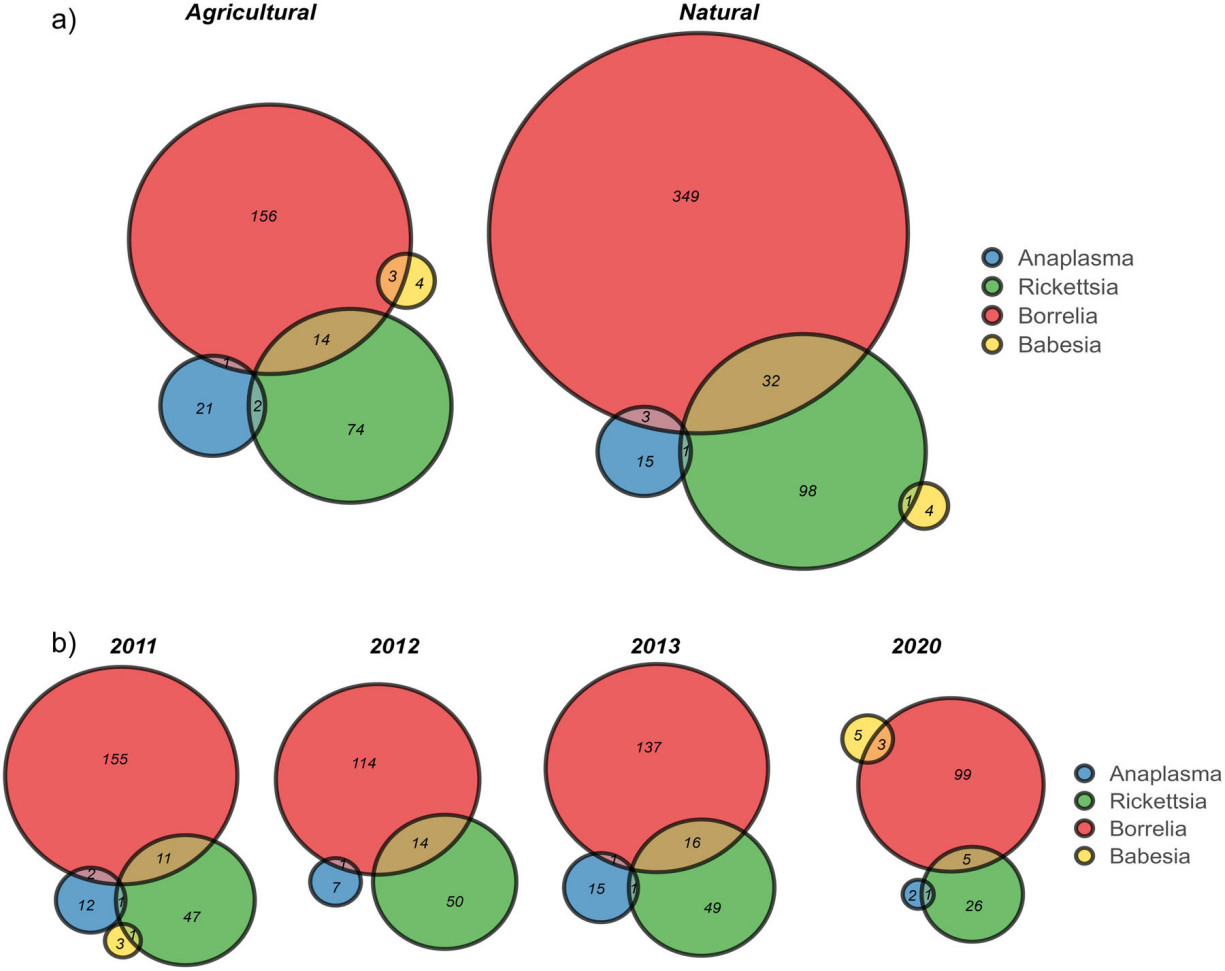
Number of co‐infections identified in *I. ricinus* ticks. Panel a: by habitat type (agricultural or natural); Panel b: across sampling years (2011, 2012, 2013, 2020).

**Figure G1:**
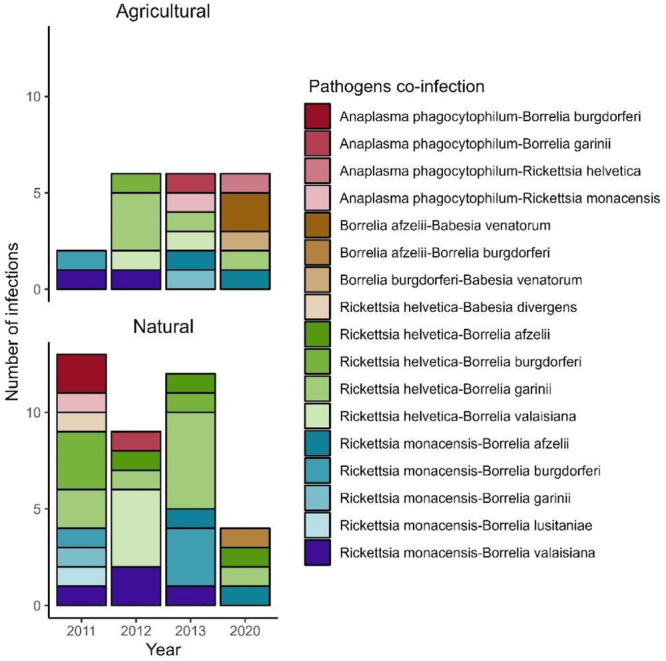
Number and detail of co‐infections identified in the *Ixodes ricinus* ticks in the two habitat types (agricultural and natural) and across the sampling years (2011–2013, 2020). The color gradients show the coexistence between multiple tick‐borne pathogens carried by the collected ticks.

In total 58 ticks were coinfected with 116 double infections, representing 8% of the positive ticks (58/720) or 2.2% of all ticks tested (58/2652). The number of coinfected ticks was higher in natural (*N* = 38) compared to agricultural habitats (*N* = 20) (Figure 5a). All the combinations of pathogens were maintained across habitat types except for *Babesia* spp. which was associated with *B. burgdorferi* s.l. in agricultural habitats, and with *Rickettsia* spp. in natural ones. When considering multiple associations between TBP species (Figure G1 for details), the most frequent association was between *Rickettsia* (*R. helvetica* and *R. monacensis*) and *Borrelia* species (*B. afzelii*, *B. garinii*, *B. burgdorferi* s.s., *B. lusitaniae* and *B. valaisiana*) with 1.7% (46/2652) ticks, mostly found in natural habitats (*N* = 32). Especially *R. helvetica* was found to be associated with four out of five *B. burgdorferi* s.l. (*N* = 28). *R. monacensis* was identified with *B. burgdorferi* s.l. (*N* = 18) and once in association with *B. lusitaniae* (*N* = 1). Other co‐infections included *A. phagocytophilum* with *B. burgdorferi* s.s. (*N* = 2), *B. garinii* (N = 2), *R. helvetica* (*N* = 1) and *R. monacensis* (N = 2). *Babesia* spp. was associated with *B. afzelii* (*N* = 2), *B. burgdorferi* s.s. (*N* = 1) and *R. helvetica* (*N* = 1). Finally, a co‐infection between *B. afzelii* and *B. burgdorferi* s.s. was also identified (*N* = 1).

Regarding the temporal pattern, pathogens were associated slightly differently across sampling years (see Figure 5b). *Babesia* spp. was associated with *Rickettsia* spp. in 2011, while they coinfected ticks with *B. burgdorferi* s.l. in 2020. Moreover, *A. phagocytophilum* was solely bound with *B. burgdorferi* s.l. in 2012 and with *Rickettsia* spp. in 2020, while in 2011 and 2013, it was found together with both the above‐mentioned bacteria (Figure 5).

## 4 Discussion

In this study, we examined the composition of the TBP community and their infection rates in the Autonomous Province of Trento in the northeastern Italian Alps. This area proved to be an excellent study system given the diffuse human activities in the natural landscapes, and the various studies carried out to date to investigate the eco‐epidemiological processes involving *I. ricinus* as a vector of zoonotic diseases (Mantelli et al. 2006; Cagnacci et al. 2012; Rosso et al. 2017; Baráková et al. 2018; Rosà et al. 2018, 2019; Marini et al. 2023). It is of paramount importance to provide early warning of the rise in hazards and newly emerging pathogens, already exacerbated by rapid climate and land use changes (Diuk‐Wasser, VanAcker, and Fernandez 2021; Gilbert 2021).

Compared to previous studies, we performed a more in‐depth molecular analysis, which allowed us to identify the pathogens at the species level, including co‐infections. We then evaluated the spatiotemporal variation of zoonotic TBPs in the area after a 10‐year interval, considering different habitat types and potential changes in host communities.

We reported the circulation of 11 species of bacterial and protozoan agents belonging to *B. burgdorferi* s.l. complex, *Anaplasma*, *Rickettsia*, and *Babesia*, all considered hazardous for human health; and the occurrence of co‐infections due to their relevance for public health. As expected from previous studies in the area (Rosà et al. 2018), pathogens occurrence showed heterogeneity in their distribution according to the type of habitat.

Among the TBDs investigated in this study, Lyme disease (or Lyme borreliosis) is currently the most common arthropod‐borne disease in temperate regions of the northern hemisphere, causing global public health problems (Steinbrink et al. 2022). Compared to our previous study that considered the pathogen at the genus level (Rosà et al. 2018), we identified 5 genotypes of *B. burgdorferi* s.l. complex. *B. lusitaniae* has been occasionally detected in human patients (Collares‐Pereira et al. 2004; Lopes de Carvalho et al. 2008), while the pathogenicity of *B. valaisiana* in humans is currently under debate (Diza et al. 2004; Margos, Sing, and Fingerle 2017). Meanwhile, the other three detected species (*B. burgdorferi* s.s., *B. afzelii* and *B. garinii*) are well‐recognized Lyme borreliosis‐causing agents (Strnad and Rego 2020). The overall infection rate of *B. burgdorferi* s.l. was higher when compared to data from a European review (12.3%; Strnad et al. 2017) and a recent study conducted in the Eastern Italian Alps (0.2%–6.6%; Bertola et al. 2021). The increase was even more profound when compared to a study performed in the same area 20 years ago (1.32%; Mantelli et al. 2006), although this was probably due to methodological limitations. Other studies conducted in the Alps and the Apennines (Capelli et al. 2012; Ragagli et al. 2016; Oechslin et al. 2017; Millet et al. 2019; Garcia‐Vozmediano et al. 2020; Schötta et al. 2023) reported a prevalence similar to ours. We recorded an increase in prevalence rates from nymph to adult ticks in *B. lusitaniae* and *B. burgdorferi* s.s., and in natural habitats, highlighting the complex relationship between host competence and habitat types. The complexity of hosts’ competence for *B. burgdorferi* s.l. emerged indirectly from the high prevalence of *B. lusitaniae* and *B. burgdorferi* s.s. in natural compared to agricultural habitats. Landscape acts as an important driver of *B. burgdorferi* s.l. persistence by affecting the movement and community composition of hosts, as well as ticks’ activity and their abundance (Millins et al. 2018; Diuk‐Wasser, VanAcker, and Fernandez 2021). Given the highly diversified host community on which *B. burgdorferi* s.l. relies on (Hofmeester et al. 2016; Wolcott et al. 2021), woodland landscapes can provide suitable conditions for vertebrate hosts (such as rodents, reptiles, birds, and large mammals like deer) involved in the bacterium sylvatic cycle (Millins et al. 2017). Among others, two studies showed evidence of the relative importance of woodland habitats for the risk of Lyme disease, with higher prevalence and density of infected ticks in woodland landscapes with respect to adjacent open habitats (Halos et al. 2010; Gilbert 2016).

Conversely, *A. phagocytophilum* and *Ba. venatorum* resulted more associated with agricultural habitats. Several terrestrial vertebrate species are considered hosts for *A. phagocytophilum*, such as foxes, wild boars, birds, reptiles, and large wild‐living ruminants (Stuen, Granquist, and Silaghi 2013), although depending on *A. phagocytophilum* ecotypes (Baráková et al. 2014; Jahfari et al. 2014; Jaarsma et al. 2019). Wild ungulates, such as roe deer, red deer, and mountain ungulates are hosts also for *Ba. venatorum* (Yabsley and Shock 2013). In particular, roe deer, one of the main ungulate species found in the study area, exhibits marked behavioral and ecological plasticity. It can exploit not only ecotonal and forested areas, but also human‐modified landscapes including open agricultural ones (Tinoco Torres et al. 2011), especially during spring and autumn (De Groeve et al. 2023), overlapping thereafter with the peak season of ticks. Moreover, although in the early 1970s population abundance of wild ungulates, such as roe deer and red deer, generally increased in the Province of Trento (Sieff 2020; Passoni et al. 2023), in recent years red deer showed a steep increment, while roe deer remained rather stable or slightly decreased (Sieff 2020). In this way, the significant use of agricultural habitats by this ungulate species, combined with the fluctuations in their abundance, may have driven infection rates with *A. phagocytophilum* and *Ba. venatorum*, confirming (Hamšíková et al. 2019) and (Overzier et al. 2013). The pivotal role of roe deer as a competent host for *A. phagocytophilum* is supported by the increasing prevalence of this microorganism detected in all tick stages from nymphs to adults (Stuen, Granquist, and Silaghi 2013; Jensen et al. 2017). On the other side, since the ecotype of *A. phagocytophilum* associated with *I. ricinus* was linked with deer, rather than with rodents (Blaňarová et al. 2014; Rosso et al. 2017), its decrease in prevalence in 2020 may reflect the trend of declining roe deer population. This was also observed in Germany (Glass, Springer, and Strube 2022). Indeed, the temporal variation of roe deer abundance may have diverted infected *I. ricinus* ticks (Rifkin, Nunn, and Garamszegi 2012), thereby decreasing the prevalence of the bacterium in questing ticks. In general, the observed infection rates of *Babesia* spp. and of the two detected species (*Ba. divergens* and *Ba. venatorum*) confirmed the results previously obtained in Italy (Capelli et al. 2012; Castro et al. 2015) and in other Alpine countries, like Switzerland (Gigandet et al. 2011; Oechslin et al. 2017) and France (Jouglin et al. 2017), while higher prevalence was observed in Austria (2.7%; Schötta et al. 2017).

*Rickettsiae* spp. prevalence did not show any significant influence on habitat types or years. Our results are similar or lower with respect to previous studies from Austria (3.8‐13.3%; Schötta et al. 2023), France (4.6‐17.6%; Halos et al. 2010; Akl et al. 2019) and Poland (15.0%; Zając et al. 2023). Compared with Italian data, our results lay among the highest values reported from the Western Italian Alps (13.3‐20.7%; Millet et al. 2019; Garcia‐Vozmediano et al. 2020) and are the lowest reported in the Eastern Italian Alps (0.3%–3.7%; Bertola et al. 2021), suggesting a sort of a west‐to‐east decreasing gradient. Interestingly, although the prevalence (0.43%) was low in 2011, we found evidence of *R. slovaca*. This species is usually carried by *Dermacentor* spp. ticks due to microbial interference (Cutler et al. 2021), although laboratory findings proved the potential transmission between *Dermacentor* spp. and *I. ricinus* on the same host via co‐feeding (Bartosik et al. 2017). The bacterium of *R. slovaca* is associated with a newly recognized atypical rickettsiosis (scalp eschar and neck lymphadenopathy after tick bite, SENLAT). Our detection in *I. ricinus*, together with another work from Austria (Schötta et al. 2017), may suggest a new potential health risk in the Alpine area, as its incidence has likely been underestimated to date (Parola et al. 2009; Del Giudice et al. 2023).

Only recently, the co‐infection patterns within ticks have been investigated (Civitello, Rynkiewicz, and Clay 2010), thanks to the availability of molecular diagnostic tools. This allowed us to highlight the presence of multiple pathogens and endosymbionts in individual tick samples, which might affect disease risk (Diuk‐Wasser, Vannier, and Krause 2016; Cutler et al. 2021). In this study, we identified several co‐infections within ticks, especially in natural habitats (forests), which confirms the potential involvement of a broad variety of wildlife‐competent species serving as hosts for more than one pathogen (Carpi et al. 2011; Noden, Roselli, and Loss 2022). Among co‐infections, *B. burgdorferi* s.l. and *Rickettsia* spp. formed the most frequent association. Although the incidence was lower, this was consistent with other studies (Lommano et al. 2012; Raileanu et al. 2017; Raulf et al. 2018). Remarkably, Raulf et al. (2018) provided evidence that this association may favor higher replication rates of these two pathogens in the tick, but at the same time have a detrimental influence on the tick vector itself, that is, increasing mortality of nymphs. Moreover, we observed co‐infections also among different *Borrelia* genotypes, that is, *B. afzelii* and *B. burgdorferi s.s*. as found in Moutailler et al. (2016). Further, we detected co‐infections of *A. phagocytophilum* with *B. burgdorferi* s.l. and with *Rickettsia* spp. confirming that *I. ricinus* may facilitate the synergies between *A. phagocytophilum* and other pathogens (Civitello, Rynkiewicz, and Clay 2010). The interactions involving *Babesia* spp. resulted instead rare, although the co‐occurrence between *B. microti* and *B. burgdorferi* s.l. in the United States is more frequent and can exacerbate and prolong disease symptoms in humans (Diuk‐Wasser, Vannier, and Krause 2016). Babesiosis is an emerging zoonotic tick‐borne parasitic disease (Hildebrandt and Hunfeld 2014) whose reservoirs are small mammals and is present in the United States (Swanson et al. 2023) and in Europe (Silaghi et al. 2012). The synergism between *Borrelia* and *Babesia* spp. has been rarely detected in Europe (Lommano et al. 2012), but recently it has been proved that *Babesia* infection tended to occur more frequently among *Borrelia*‐positive ticks (Pawełczyk et al. 2021). In this study, *Ba. venatorum* was associated with *R. helvetica* in natural habitats in 2011, while we found it more frequently with two different species of the *Borrelia* complex (*B. afzelii* and *B. burgdorferi* s.s.) in agricultural habitats in 2020. Even though we did not find *B. microti* in questing ticks, a previous study performed in the same area identified *B. microti* from *I. ricinus* ticks collected from rodents (Baráková et al. 2018). This can pose a threat to human health and should be considered when diagnosing and treating tick bite symptoms.

This study describes the TBP community and the disease risk in an Alpine area where human exposure to these pathogenic microorganisms is likely enhanced by recreational and leisure activities. We underlined that spatiotemporal variability in pathogens' distribution and associations is dynamic and in constant evolution, being driven among others by a combination of land use patterns, and wildlife host communities (Parham et al. 2015; Keesing and Ostfeld 2021). Additionally, vectors, host competence, immunity, and the coexistence of multiple pathogens within one host, add another layer of complexity to this relationship (Downs et al. 2019; Cutler et al. 2021). We provided important insights into the occurrence and prevalence of TBPs in an Alpine area, including multiple pathogen‐host associations, which occur or may soon emerge in this study system. This provides useful knowledge that, even if sustained by limited spatio‐temporal replications, suggests the importance of long‐term community‐based studies applying a multidisciplinary “One Health” approach.

## Author Contributions

**Fausta Rosso:** conceptualization, methodology, investigation, resources, writing–original draft, writing–review and editing. **Giulia Ferrari:** conceptualization, methodology, formal analysis, data curation, writing–original draft, writing–review and editing. **Tobias Weil:** formal analysis, data curation, writing–review and editing. **Valentina Tagliapietra:** conceptualization, methodology, investigation, resources, writing–review and editing. **Giovanni Marini:** methodology, formal analysis, writing–review and editing. **Francesca Dagostin:** methodology, writing–review and editing. **Daniele Arnoldi:** investigation, writing–review and editing. **Matteo Girardi:** methodology, investigation, writing–review and editing. **Annapaola Rizzoli:** conceptualization, methodology, resources, writing–review and editing. All authors have read and agreed to the published version of the manuscript.

## Ethics Statement

The authors have nothing to report.

## Conflicts of Interest

The authors declare no conflicts of interest.

## Data Availability

The data generated and analyzed during this study are available via NCBI, with accession numbers listed at the end of the manuscript. Sequence data are available via NCBI. The accession numbers for the sequences are Borreliella valaisiana (OR709028–OR709151), Borreliella lusitaniae (OR709005–OR709027), Borreliella garinii (OR709152–OR709322), Borreliella burgdorferi (OR709323–OR709433), Borreliella afzelii (OR709434–OR709562), Rickettsia helvetica_17kDa (OR819468–OR819604), Rickettsia monacensis_F9 (OR819605), Rickettsia monacensis_OmpA (OR819606–OR819688), Rickettsia slovaca (OR819689), Babesia venatorum (OR791120–OR791126), Babesia divergens (OR783373–OR783377), Anaplasma phagocytophilum (OR783196–OR783238).

## Appendix A Polymerase Chain Reaction Details

**Table A1:** List of target genes and specific F and R primers for detection of *Babesia* spp., *Borrelia* spp., *Anaplasma* spp., and *Rickettsia* spp. from questing *Ixodes ricinus* ticks.

Pathogen	Target gene	Primer F	Primer R
*Babesia* spp.	18S ribosomal RNA gene (430 bp)	BN2 5′–TAGTTTATGGTTAGGACTACG– 3'	BJ1 5′–GTCTTGTAATTGGAATGATGG – 3'
*Anaplasma* spp. 1 PCR	16S rRNAgene (546 bp)	ge3a 5′–CACATGCAAGTCGAACGGATTATT C–3'	ge10r 5′–TTCCGTTAAGAAGGATCTAATCTC C– 3'
*Anaplasma* spp. 2 PCR		ge9f 5′–AACGGATTATTCTTTATAGCTTGCT–3'	ge2 5′–GGCAGTATTAAAAGCAGCTCCAGG–3'
*Rickettisa* spp. 1 PCR	17 kDa common antigen (450 bp)*	Rr17K.1p 5′–TTTACAAAATTCTAAAAACCAT–3'	Rr17K.539n 5′–TCAATTCACAACTTGCCATT–3'
*Rickettsia* spp. 2 PCR		Rr17K.90p 5′–– 3'	Rr17K.539n 5′–TCAATTCACAACTTGCCATT–3'
*Borrelia* spp. 1 PCR	5S‐23S ribosomal RNA intergenic spacer (230 bp)	23SC1 5′–TAAGCTGACTAATACTAATTACCC–3'	23SN1 5′–ACCATAGACTCTTATTACTTTGACC–3'
*Borrelia* spp. 2 PCR		5SC2 5′–GAGAGTAGGTTATTGCCAGGG–3'	23SN2 5′–ACCATAGACTCTTATTACTTTGACCA–3'

*Note:* Primer F = primer forward; Primer R = primer reverse; * = Rickettsia monacensis has been investigated targeting outer membrane protein gene A (ompA) (primers: ompA13f 5′–GCAATTCAAAAAGGTCTTAAA–3′ and ompA545R 5′–TTTCCTGTAAGTGTTATCTTTG–3′; 580 bp).

**Table A2:** PCR reactions for detection of *Babesia* spp., *Borrelia* spp., *Anaplasma* spp., and *Rickettsia* spp. from questing *Ixodes ricinus* ticks.

Pathogen	Final volume (μL)	DNA volume (μL)	H2O (μL)	Reaction buffer (μL)	25 mM MgCl2 (μL)	10 mg/mL BSA (μL)	10 pmol/μL Primer F (μL)	10 pmol/μL Primer R (μL)	10 mM dNTPs (μL)	5 U/μL Taq (μL)
*Babesia* spp.	50.0	2.0	28.75	10.0	4.0	—	2.0	2.0	1.0	0.25
*Anaplasma* spp. 1 PCR	50.0	2.0	30.75	10.0	4.0	—	1.0	1.0	1.0	0.25
*Anaplasma* spp. 2 PCR	50.0	2.0	30.75	10.0	4.0	—	1.0	1.0	1.0	0.25
*Rickettsia* spp. 1 PCR	50.0	1.0	28.5	5.0	5.0	1.0	4.0	4.0	1.25	0.25
*Rickettsia* spp. 2 PCR	50.0	2.0	23.5	5.0	4.0	1.0	4.0	4.0	1.25	0.25
*Borrelia* spp. 1 PCR	25.0	5.0	13.75	2.5	2.0	0.0	0.5	0.5	0.5	0.25
*Borrelia* spp. 2 PCR	25.0	5.0	13.7	2.5	2.5	0.0	0.3	0.3	0.5	0.2

*Note:* — = represents reagent not included.

**Table A3:** Temperature cycling profile of PCR for detection of *Babesia* spp., *Borrelia* spp., *Anaplasma* spp., and *Rickettsia* spp. from questing *Ixodes ricinus* ticks.

Pathogen	Initial temperature (°C)	Denaturation temperature (°C)	Annealing temperature (°C)	Extension temperature (°C)	Final temperature (°C)	Number of cycles
*Babesia* spp.	95 × 2'	95 × 20″	53 × 30″	72 × 40″	72 × 5'	40
*Anaplasma* spp. 1 PCR	95 × 2'	95 × 20″	54 × 30″	72 × 40″	72 × 5'	35
*Anaplasma* spp. 2 PCR	95 × 2'	95 × 20″	54 × 30″	72 × 40″	72 × 5'	40
*Rickettsia* spp. 1 PCR	94 × 3'	94 × 30″	56 × 30″	72 × 1'	72 × 7'	15
*Rickettsia* spp. 2 PCR	94 × 3'	94 × ″	57 × 35″	72 × 1'10'	72 × 7'	35
*Borrelia* spp. 1 PCR	94 × 10'	94 × 30″	57 × 30″	72 × 1'	72 × 7'	45
*Borrelia* spp. 2 PCR	94 × 3'	94 × 30″	52 × 30″	72 × 1'	72 × 7'	25

## Appendix D Shannon Index

**Table D1:** Tick‐borne pathogens' diversity based on the Shannon Index in the two habitat types (agricultural and natural) and across years (Province of Trento, 2011–2013, 2020).

Habitat	Year	Shannon index
Agricultural	2011	2.075.425
Agricultural	2012	1.699.871
Agricultural	2013	1.847.041
Agricultural	2020	2.075.052
Natural	2011	2.009.177
Natural	2012	1.847.591
Natural	2013	1.918.951
Natural	2020	1.939.258

## Appendix F Binomial Generalized Linear Models (GLMs) of Tick‐Borne Pathogens' Prevalence

**Table F1:** Coefficients of the models fitting the prevalence of 11 pathogens in ticks across the sampling years (2011–2013, 2020), depending on habitat type.

Model	Pathogen	Predictors	Coefficients	S.E.	CI	*p*
1	Anaplasma phagocytophilum	(Intercept)	−3.79 ***	0.21	−4.22 to −3.41	< 0.001
		Habitat [Natural]	−0.61 *	0.31	−1.23 to −0.01	0.047
2	Babesia divergens	(Intercept)	−6.99 ***	1.00	−9.85 to −5.50	< 0.001
		Habitat [Natural]	1.02	1.12	−0.90–3.99	0.363
3	Babesia venatorum	(Intercept)	−5.19 ***	0.41	−6.11 to −4.48	< 0.001
		Habitat [Natural]	−2.17 *	1.08	−5.11 to −0.40	0.045
4	Borrelia afzelii	(Intercept)	−3.09 ***	0.15	−3.40 to −2.81	< 0.001
		Habitat [Natural]	0.20	0.19	−0.17–0.57	0.298
5	Borrelia burgdorferi	(Intercept)	−3.83 ***	0.21	−4.27 to −3.44	< 0.001
		Habitat [Natural]	1.02 ***	0.24	0.57–1.51	< 0.001
6	Borrelia garinii	(Intercept)	−2.82 ***	0.13	−3.09 to −2.57	< 0.001
		Habitat [Natural]	0.23	0.16	−0.09–0.56	0.156
7	Borrelia lusitaniae	(Intercept)	−6.29 ***	0.71	−8.09 to −5.16	< 0.001
		Habitat [Natural]	1.99 **	0.74	0.76–3.83	0.007
8	Borrelia valaisiana	(Intercept)	−3.24 ***	0.16	−3.56 to −2.94	< 0.001
		Habitat [Natural]	0.35	0.20	−0.03–0.74	0.073
9	Rickettsia helvetica	(Intercept)	−2.87 ***	0.13	−3.15 to −2.62	< 0.001
		Habitat [Natural]	−0.07	0.18	−0.41–0.29	0.714
10	Rickettsia monacensis	(Intercept)	−3.52 ***	0.18	−3.90 to −3.19	< 0.001
		Habitat [Natural]	0.17	0.23	−0.27–0.63	0.457
11	Rickettsia slovaca	(Intercept)	−6.99 ***	1.00	−9.85 to −5.50	< 0.001
		Habitat [Natural]	−22.14	32329.65	NA–7058.81	0.999

*Note:* “Habitat” = habitat type (agricultural or natural; reference category: agricultural); “CI” = confidence interval; “*p*” = *p*‐value. Significance level: **p* < 0.05, ***p *< 0.01, *** *p* < 0.001.

**Table F2:** Coefficients of the models fitting the prevalence of 11 pathogens in ticks across the sampling years (2011–2013, 2020), depending on the tick stage.

Model	Pathogen	Predictors	Coefficients	S.E.	CI	*p*
1	Anaplasma phagocytophilum	(Intercept)	−3.30 ***	0.28	−3.90 to −2.79	< 0.001
		Age [Nymph]	−1.03 **	0.34	−1.66 to −0.33	0.002
2	Babesia divergens	(Intercept)	−25.63	11660.42	−5116.29 to −8059.88	0.998
		Age [Nymph]	19.50	11660.42	−1315.99–NA	0.999
3	Babesia venatorum	(Intercept)	−5.89 ***	1.00	−8.76 to −4.41	< 0.001
		Age [Nymph]	−0.05	1.08	−1.82–2.89	0.966
4	Borrelia afzelii	(Intercept)	−3.22 ***	0.27	−3.80 to −2.72	< 0.001
		Age [Nymph]	0.28	0.29	−0.25–0.89	0.332
5	Borrelia burgdorferi	(Intercept)	−2.96 ***	0.24	−3.47 to −2.51	< 0.001
		Age [Nymph]	−0.19	0.26	−0.69–0.35	0.462
6	Borrelia garinii	(Intercept)	−2.65 ***	0.21	−3.09 to −2.26	< 0.001
		Age [Nymph]	−0.03	0.23	−0.46–0.44	0.903
7	Borrelia lusitaniae	(Intercept)	−3.80 ***	0.36	−4.58 to −3.16	< 0.001
		Age [Nymph]	−1.23 **	0.44	−2.07 to−0.31	0.006
8	Borrelia valaisiana	(Intercept)	−3.02 ***	0.25	−3.54 to−2.56	< 0.001
		Age [Nymph]	0.00	0.27	−0.50 – 0.56	0.996
9	Rickettsia helvetica	(Intercept)	−2.74 ***	0.22	−3.20 – −2.34	< 0.001
		Age [Nymph]	−0.20	0.24	−0.65–0.30	0.416
10	Rickettsia monacensis	(Intercept)	−3.15 ***	0.26	−3.71 to −2.67	< 0.001
		Age [Nymph]	−0.32	0.29	−0.87–0.28	0.265
11	Rickettsia slovaca	(Intercept)	−25.63	11660.42	−5116.29 to −8059.88	0.998
		Age [Nymph]	17.89	11660.42	−2556.51–NA	0.999

*Note:* “Age” = tick stage (nymph or adult; reference category: adult); “CI” = confidence interval; “p” = *p*‐value. Significance level: * *p* < 0.05, ***p* < 0.01, ****p* < 0.001.
